# Highly Pathogenic Avian Influenza A(H5N8) Clade 2.3.4.4b Viruses in Satellite-Tracked Wild Ducks, Ningxia, China, 2020

**DOI:** 10.3201/eid2805.211580

**Published:** 2022-05

**Authors:** Xinru Lv, Xiang Li, Heting Sun, Yi Li, Peng Peng, Siyuan Qin, Weidong Wang, Yuecheng Li, Qing An, Tian Fu, Fengyi Qu, Qiuzi Xu, Rongxiu Qin, Zhenliang Zhao, Meixi Wang, Yulong Wang, Yajun Wang, Xiangwei Zeng, Zhijun Hou, Chengliang Lei, Dong Chu, Yanbing Li, Hongliang Chai

**Affiliations:** Northeast Forestry University College of Wildlife and Protected Area, Harbin, China (X. Lv, X. Li, Yi Li, Q. An, T. Fu, F. Qu, Q. Xu, R. Qin, Z. Zhao, M. Wang, Yulong Wang, Yajun Wang, X. Zeng, Z. Hou, H. Chai);; National Forestry and Grassland Administration, Shenyang, China (H. Sun, P. Peng, S. Qin, C. Lei, D. Chu);; Monitoring Center for Terrestrial Wildlife Epidemic Diseases, Yinchuan, China (W. Wang, Yuecheng Li);; Harbin Veterinary Research Institute, Harbin (Yanbing Li)

**Keywords:** influenza, highly pathogenic avian influenza A(H5N8) viruses, influenza virus, viruses, H5N8, subtype, clade 2.3.4.4b, respiratory infections, birds, wild ducks, satellite tracked, phylogeny, epidemic, public health, zoonoses, Ningxia, China

## Abstract

During October 2020, we identified 13 highly pathogenic avian influenza A(H5N8) clade 2.3.4.4b viruses from wild ducks in Ningxia, China. These viruses were genetically related to H5N8 viruses circulating mainly in poultry in Europe during early 2020. We also determined movements of H5N8 virus‒infected wild ducks and evidence for spreading of viruses.

A novel reassortant highly pathogenic avian influenza (HPAI) A(H5N8) virus belonging to clade 2.3.4.4 was detected in poultry and wild birds in South Korea during January 2014 (*1*) and spread rapidly by migration of wild birds to Asia, Europe, and North America (*2*). Clade 2.3.4.4 HPAI H5N8 viruses caused additional influenza outbreaks worldwide during 2016 and continued circulating in birds in Asia, Europe, and Africa (*3**–**5*).

In October 2020, clade 2.3.4.4b HPAI H5N8 viruses were detected in wild swans in China (*6*). A clade 2.3.4.4b H5N8 virus infection in humans was reported in Russia during December 2020, indicating a possible increased risk for these viruses crossing species barriers (*7*). In this study, we investigated the emergence of HPAI H5N8 viruses in wild ducks in Ningxia, in western China, during October 2020 and performed satellite tracking to determine the flyways of wild ducks.

## The Study

Ningxia, located at the intersection of the Central Asian and East Asian-Australasian Flyways, is an ideal location for influenza surveillance. We collected 275 paired oropharyngeal and cloacal swab specimens from net-caught wild ducks at the Changshantou Reservoir in Ningxia (37°16′14′′N, 105°43′5′′E) during October 2020. We inoculated all samples into 10-day-old, embryonated, specific pathogen–free chicken eggs for virus isolation. Thirteen samples were positive for H5N8 subtype avian influenza virus (AIV) by reverse transcription PCR. We sequenced full-length genomes and submitted them to the GISAID EpiFlu database (https://www.gisaid.org) (Appendix Table 1).

We attached solar-powered global positioning system satellite trackers to 12 apparently healthy mallards (*Anas platyrhynchos*) at the capture site and released the birds immediately. We successfully obtained movement tracks for 9 mallards to identify their wintering and stopover sites. We isolated H5N8 viruses from 2 of the satellite-tracked mallards (birds NX-175 and NX-176), but the remaining 7 mallards were negative for AIV (Figure; Appendix Table 2, Figures 1, 2).

**Figure:**
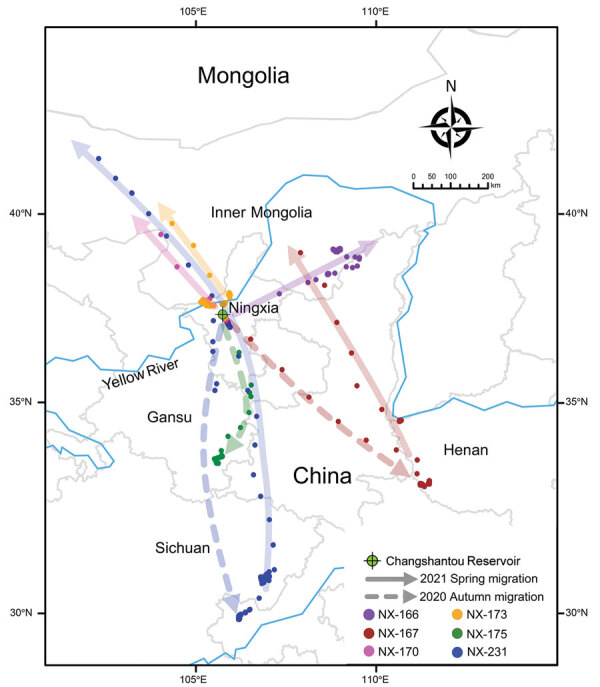
Migratory routes of 6 of 9 successfully satellite-tracked mallards infected with highly pathogenic avian influenza A(H5N8) clade 2.3.4.4b viruses, Ningxia, China, 2020. Mallards are indicated by different colors. The sampling site (Changshantou Reservoir) is indicated. Solid and dashed lines indicate spring migration in 2021 and autumn migration in 2020, respectively. Because the other 3 successfully satellite-tracked mallards (birds NX-169, NX-174, and NX-176) had been moving around the sampling point, their movements are not shown.

All H5N8 isolates were identified as HPAIVs by the amino acid sequence REKRRKR/GLF at the hemagglutinin (HA) cleavage site. H5 phylogenetic analysis classified Ningxia isolates into clade 2.3.4.4b and divided them into 2 distinct groups according to tree topology (Appendix Figure 3). Most isolates (n = 12) shared high nucleotide identities in 8 gene segments (99.6%–100%) with viruses responsible for disease outbreaks in poultry in Europe during early 2020; these segments were closely related to H5N8 viruses from South Korea and Japan, isolated in October and November 2020 and recognized as subclade 2.3.4.4b1, (Appendix Table 3). The remaining isolate, A/common teal/Ningxia/105/2020(H5N8) (from mallard NX-105), clustered with HPAI H5 viruses that were prevalent in Eurasia in autumn 2020 and recognized as subclade 2.3.4.4b2. A similar tree topology was shown in all 8 segments of Ningxia virus isolates (Appendix Figure 4). Mallard isolate NX-105 and the human isolate A/Astrakhan/3212/2020(H5N8) (human H5N8) from Russia had relatively high nucleotide identities of 99.2%–99.8% in 8 gene segments.

Bayesian phylogenetic analysis showed that the most recent common ancestor of the genome of isolate NX-105 and its neighbor strains emerged during June‒October 2020. Ningxia b1 isolates emerged during August‒September 2020, and East Asian lineage (b1 viruses including Ningxia subclade 2.3.4.4b1 isolates, strains from Japan and South Korea) emerged at the genome level during May‒August 2020 (Appendix Table 4, Figure 5).

Several amino acid mutations in the HA protein (H5 numbering) were associated with increased binding to human‐like receptor (α‐2,6‐sialic acid) (*8**–**11*). Both Ningxia H5N8 isolates and the human H5N8 isolate from Russia had the S133A and T156A mutations, and isolate NX-105 had extra T188I and V210I substitutions, suggesting that this isolate might be more adaptable at infecting humans than the human H5N8 virus. All isolates lacked the Q222L and G224S mutations in the HA protein, including the human H5N8 virus, and lacked the mammalian adaptation markers Q591K, E627K, and D701N mutations in the polymerase basic 2 protein (*12*). Both Ningxia H5N8 isolates and the human H5N8 virus also had other molecular markers associated with increased virulence and transmission among mammals (Appendix Table 5).

Satellite tracking showed that 2 mallards (NX-167, negative for AIV, and NX-175, infected with H5N8 virus) migrated to the wintering ground without a long duration in Ningxia. Mallard NX-167 flew directly to Henan at a high speed (82.1–116.2 km/h). In contrast, mallard NX-175 showed a greatly decreased speed (34.1–61.8 km/h) after a short stopover at the junction of Ningxia and Gansu, and eventually reached Gansu (Appendix Figure 1). Another H5N8-infected mallard (NX-176) had been moving around the sampling site until we lost the tracking signals on December 25, 2020 (Appendix Figure 2). These results indicated that mallards could continue to migrate after being infected with HPAI H5N8 viruses, but their movements would be affected.

## Conclusions

Previous studies have demonstrated a key role for wild waterfowl in the continental transmission of HPAIVs (*13*). In this study, we inferred that H5N8 viruses emerging in Ningxia were likely to be transmitted by migration of infected wild ducks. H5N8 virus outbreaks occurred in the poultry industry in Europe during spring 2020, and the responsible viruses might have been introduced into the wild-bird gene pool through contact with infected poultry (*14*). Wild ducks are short-distance migratory birds, which generally find it difficult to migrate directly from Europe to eastern Asia. Strains from eastern Asia had high nucleotide identity (99.3%–100%) at the genome level, indicating that subclade 2.3.4.4b1 H5N8 viruses might be maintained at common breeding and stopover sites of wild ducks that winter in China, Japan, and South Korea.

The long branch lengths for all segments of the East Asian lineage compared with those for strains from Europe suggested that the virus had been circulating undetected for the intervening period and seemed to have a common ancestor from older viruses during early 2020 or 2019 (Appendix Figure 4). A previous study of the origin of clade 2.3.4.4b HPAI H5N6 viruses isolated in wild ducks in Ningxia in 2017 indicated a similar transmission pattern (*15*). In addition, isolate NX-105 showed an extremely close phylogenetic relationship with the 2020 isolates from Russia (Appendix Figure 4), which also seemed to be transmitted to China by migratory wild ducks.

The movement of mallard NX-175 proved that mallards infected with HPAI H5N8 viruses could continue to migrate, resulting in potential wide spreading of HPAI H5N8 viruses (Appendix Figure 1). Satellite tracking showed that continuous and stable tracking signals for 3 mallards (NX-170, NX-173, and NX-231) migrating northward during April 2021 were suddenly lost during a high-speed flight in Inner Mongolia (Figure). Assuming no damage to the transmitters, we inferred that these 3 mallards had already flown out of China for breeding, and we will therefore not receive additional signals from overseas until the birds return to China during their autumn migration. Further satellite tracking studies are being performed to determine the breeding and stopover grounds in northern Ningxia, China, as essential means of tracing the origins of AIVs and providing future early warnings for these viruses.

Ningxia H5N8 virus isolates showed highly similar mutations to those of human H5N8 viruses, and isolate NX-105 is highly homologous at the genome level, indicating that wild duck‒origin viruses could pose an increased threat to public health. Long-term surveillance of wild bird‒origin AIVs and international collaboration in AIV monitoring of migratory birds will help support early warning for influenza epidemics.

Appendix Additional information on highly pathogenic avian influenza A(H5N8) clade 2.3.4.4b viruses in satellite-tracked wild ducks, Ningxia, China, 2020.
