# Supplementary material for: Highly Pathogenic Avian Influenza A(H5N8) Clade 2.3.4.4b Viruses in Satellite-Tracked Wild Ducks, Ningxia, China, 2020
Source: Emerg Infect Dis. 2022 May;28(5):1039–42. doi: 10.3201/eid2805.211580 (PMC9045446; doi:10.3201/eid2805.211580)
Supplement: Appendix — Additional information on highly pathogenic avian influenza A(H5N8) clade 2.3.4.4b viruses in satellite-tracked wild ducks, Ningxia, China, 2020. [file 21-1580-Techapp-s1.pdf]

Article DOI: https://doi.org/10.3201/eid2805.211580

# Highly Pathogenic Avian Influenza A(H5N8) Clade 2.3.4.4b Viruses in Satellite-Tracked Wild Ducks, Ningxia, China, 2020

## Appendix

## Materials and Methods

### Samples and Virus Isolation

A total of 275 paired oropharyngeal and cloacal swab specimens were collected from net-caught wild ducks at Changshantou Reservoir, Ningxia, western China (N37°16′14″, E105°43′5″), in October 2020. The samples were oscillated and then centrifuged, and the collected supernatant was inoculated into 10-day-old specific-pathogen-free chicken embryos (National Poultry Laboratory Animal Resource Center, Harbin Veterinary Research Institute, Chinese Academy of Agriculture Sciences, Harbin, China). After 72 hours of incubation, the allantoic fluid was harvested, and the hemagglutinin (HA) activity was assayed. HA-positive isolates were further subtyped by hemagglutinin inhibition and neuraminidase inhibition assays. Viral RNA was extracted from HA-positive allantoic fluid by using QIAamp Viral RNA Mini Kit (QIAGEN, https://www.qiagen.com), reverse transcribed by using the primer Un12 and subjected to reverse transcription PCR by using the method of the World Health Organization.

**Genome Sequencing**

The PCR products of 8t fragments of the Ningxia H5N8 isolates were sequenced by using a set of specific sequencing primers previously reported (*1*). The sequence data were compiled by using the SeqMan Program (DNASTAR, https://www.dnastar.com). Thirteen H5N8 influenza viruses were isolated from 6 mallards (*Anas platyrhynchos*), 6 common teals (*A. crecca*), and 1 common pochard (*Aythya ferina*). Nucleotide sequences have been deposited in GISAID (https://www.gisaid.org) under accession nos. EPI_ISL_2815336, EPI_ISL_2815374, EPI_ISL_2820250, EPI_ISL_2820259, EPI_ISL_2820261, EPI_ISL_2820262, EPI_ISL_2820288, EPI_ISL_2820465, EPI_ISL_2820479, EPI_ISL_2820480, EPI_ISL_2820501, EPI_ISL_2820503, and EPI_ISL_2820504.

**Phylogenetic Analysis**

Homologous sequences of the Ningxia H5N8 isolates were obtained by BLAST search at the GISAID Epiflu databases in early January 2021, and 8 datasets were derived from the top 100 BLASTn hits and the genome of clade 2.3.4.4 H5N8 viruses isolated in 2020. Sequences without full alignment length were excluded. Sequences were aligned by using MAFFT (*2*) implemented in PhyloSuite version 1.2.1 (*3*). The maximum-likelihood tree of each gene was estimated by using IQ-TREE (*4*) under the best-fit substitution model for 1,0000 ultrafast bootstraps (*5*). Best-fit substitution model was selected by using the Bayesian information criterion by ModelFinder (*6*) implemented in PhyloSuite 1.21 (*3*).

For estimation of the time to the most recent common ancestor, Bayesian analysis was performed for all 8 gene segments by using BEAST version 1.10.4. (*7*). The best-fit substitution model was chosen as described above. We specified an uncorrelated lognormal relaxed clock and constant size tree prior for each segment (*8,9*). A Markov

chain Monte Carlo method was used with 50 million chain lengths to draw inference under this model. Three independent runs were combined for analyses to ensure that an adequate effective sample size (200). A maximum clade credibility tree with mean height was generated for each dataset by using TreeAnnotator version 1.10.4 (https://beast.community/treeannotator) after 10% burn-in. The maximum clade credibility trees were visualized by using FigTree version 1.4.4 (http://tree.bio.ed.ac.uk).

### Satellite Tracking

On October 15 and 16, 2020, apparently healthy mallards among the sampled birds were chosen for the satellite tracking study. We recorded bodyweight, age (adult or juvenile) and sex for each mallard before equipping them with GPS-GSM (Global Positioning System—Global System for Mobile Communications), solar-powered backpack transmitters. All mallards were released at the capture site instantly after equipping. The transmitters were obtained from Hunan Global Messenger Technology Co. Ltd., https://en.hqxs.net), and the model is HQBG1815S (http://www.hqxs.net/). The transmitters weighed 1.4%–1.5% of the mallards' body mass (average 1,032 g). Location data of the GPS transmitters were acquired from the China Mobile Communication System at 1 location in 2 h intervals by using GSM cards and were reported as latitude, longitude, and location time. The accuracy of the GPS transmitters was categorized into 5 classes: A (± 5 m), B (± 10 m), C (± 20 m), D (± 100 m), and E (± 2,000 m), and the positioning accuracy credibility is 95%. We restricted accuracy to A, B, C, and D for our analysis. We identified the wintering and stopover sites by major clusters of sequential position data, which contrasted with consistent movements and high flight speed (km/hr) during migration. This data enabled identification of the arrival and departure dates at the wintering or stopover sites, and we were able to identify the timing and duration of stopovers and migration flights, as well as the distances between stopovers. If the emitted

signals lost for >1 month, it implied that the transmitter-tagged bird was sick or dead, then the flyway monitoring was terminated.

**References**

1. Chai H. Molecular epidemiological study on influenza virus in wild birds of Heilongjiang [PhD dissertation]. Harbin (China): Northeast Forestry University; 2012.

2. Katoh K, Standley DM. MAFFT multiple sequence alignment software version 7: improvements in performance and usability. Mol Biol Evol. 2013;30:772–80. PubMed https://doi.org/10.1093/molbev/mst010

3. Zhang D, Gao F, Jakovlić I, Zou H, Zhang J, Li WX, et al. PhyloSuite: an integrated and scalable desktop platform for streamlined molecular sequence data management and evolutionary phylogenetics studies. Mol Ecol Resour. 2020;20:348–55. PubMed https://doi.org/10.1111/1755-0998.13096

4. Nguyen LT, Schmidt HA, von Haeseler A, Minh BQ. IQ-TREE: a fast and effective stochastic algorithm for estimating maximum-likelihood phylogenies. Mol Biol Evol. 2015;32:268–74. PubMed https://doi.org/10.1093/molbev/msu300

5. Minh BQ, Nguyen MA, von Haeseler A. Ultrafast approximation for phylogenetic bootstrap. Mol Biol Evol. 2013;30:1188–95. PubMed https://doi.org/10.1093/molbev/mst024

6. Kalyaanamoorthy S, Minh BQ, Wong TK, von Haeseler A, Jermiin LS. ModelFinder: fast model selection for accurate phylogenetic estimates. Nat Methods. 2017;14:587–9. PubMed https://doi.org/10.1038/nmeth.4285

7. Drummond AJ, Rambaut A. BEAST: Bayesian evolutionary analysis by sampling trees. BMC Evol Biol. 2007;7:214. PubMed https://doi.org/10.1186/1471-2148-7-214

8. Drummond AJ, Ho SY, Phillips MJ, Rambaut A. Relaxed phylogenetics and dating with confidence. PLoS Biol. 2006;4:e88. PubMed https://doi.org/10.1371/journal.pbio.0040088

9. Kingman JF. The coalescent. Stochastic Process Appl. 1982;13:235–48. https://doi.org/10.1016/0304-4149(82)90011-4

**Appendix Table 1.** Information of the thirteen H5N8 viruses isolated in Ningxia, China, October 2020

| GISAID accession no. | Isolate name | Collection date | Clade | Abbreviation |
|---|---|---|---|---|
| EPI_ISL_2815336 | A/common teal/Ningxia/105/2020(H5N8) | 2020-Oct-11 | 2.3.4.4b2 | NX-105 |
| EPI_ISL_2815374 | A/mallard/Ningxia/175/2020(H5N8) | 2020-Oct-15 | 2.3.4.4b1 | NX-175 |
| EPI_ISL_2820250 | A/mallard/Ningxia/176/2020(H5N8) | 2020-Oct-15 | 2.3.4.4b1 | NX-176 |
| EPI_ISL_2820259 | A/common teal/Ningxia/181/2020(H5N8) | 2020-Oct-15 | 2.3.4.4b1 | NX-181 |
| EPI_ISL_2820261 | A/common teal/Ningxia/189/2020(H5N8) | 2020-Oct-15 | 2.3.4.4b1 | NX-189 |
| EPI_ISL_2820262 | A/common teal/Ningxia/237/2020(H5N8) | 2020-Oct-16 | 2.3.4.4b1 | NX-237 |
| EPI_ISL_2820288 | A/mallard/Ningxia/239/2020(H5N8) | 2020-Oct-16 | 2.3.4.4b1 | NX-239 |
| EPI_ISL_2820465 | A/mallard/Ningxia/241/2020(H5N8) | 2020-Oct-16 | 2.3.4.4b1 | NX-241 |
| EPI_ISL_2820479 | A/common pochard/Ningxia/243/2020(H5N8) | 2020-Oct-16 | 2.3.4.4b1 | NX-243 |
| EPI_ISL_2820480 | A/common teal/Ningxia/245/2020(H5N8) | 2020-Oct-16 | 2.3.4.4b1 | NX-245 |
| EPI_ISL_2820501 | A/mallard/Ningxia/247/2020(H5N8) | 2020-Oct-16 | 2.3.4.4b1 | NX-247 |
| EPI_ISL_2820503 | A/mallard/Ningxia/249/2020(H5N8) | 2020-Oct-16 | 2.3.4.4b1 | NX-249 |
| EPI_ISL_2820504 | A/common teal/Ningxia/253/2020(H5N8) | 2020-Oct-16 | 2.3.4.4b1 | NX-253 |

**Appendix Table 2.** Characteristics of 9 mallards (*Anas platyrhynchos*) we successfully satellite tracked and their migration movements up to May 2021

| Bird ID | Sex (Age)* | Weight (g) | Equiping date | AIV test result | Wintering site | Arrival date at wintering site | Departure date at wintering site |
|---|---|---|---|---|---|---|---|
| NX-166 | F (A) | 1,010 | 2020-Oct-15 | Negative | Ningxia | Before 2020-Oct-15 | 2021-Mar-31 |
| NX-167 | M (A) | 1,115 | 2020-Oct-15 | Negative | Henan | 2020-Oct-21 | 2021-Mar-25 |
| NX-169 | M (A) | 1,050 | 2020-Oct-15 | Negative | Ningxia | Never left Ningxia | |
| NX-170 | M (A) | 925 | 2020-Oct-15 | Negative | Ningxia | Before 2020-Oct-15 | 2021-Apr-22 |
| NX-173 | M (A) | 1,100 | 2020-Oct-15 | Negative | Ningxia | Before 2020-Oct-15 | 2021-Apr-7 |
| NX-174 | M (A) | 1,000 | 2020-Oct-15 | Negative | Ningxia | Never left Ningxia | |
| NX-175 | F (A) | 1,060 | 2020-Oct-15 | H5N8 | Gansu | 2020-Oct-16 | |
| NX-176 | M (A) | 1,030 | 2020-Oct-16 | H5N8 | Ningxia | Never left Ningxia | |
| NX-231 | F (A) | 995 | 2020-Oct-16 | Negative | Sichuan | 2020-Dec-24 | 2021-Mar-24 |

*A, adult; AIV, avian influenza virus.

**Appendix Table 3.** Information for clade 2.3.4.4b1 isolates in this study

| Date | Location | Isolate name | Isolate ID |
|---|---|---|---|
| 2020-Oct-21 | South Korea | A/Mandarin duck/Korea/H242/2020 | EPI_ISL_631824 |
| 2020-Oct-24 | South Korea | A/Mandarin duck/Korea/K20–551–4/2020 | EPI_ISL_666687 |
| 2020-Oct-24 | Japan | A/northern pintail/Hokkaido/M13/2020 | EPI_ISL_697771 |
| 2020-Nov-09 | Japan | A/environment/Kagoshima/KU-ngr-J2/2020(H5N8) | EPI_ISL_682297 |
| 2019-Dec-30 | Poland | A/turkey/Poland/23/2019 | EPI_ISL_402134 |
| 2020-Jan-01 | Poland | A/laying_hen/Poland/002/2020 | EPI_ISL_525439 |
| 2020-Jan-02 | Poland | A/chicken/Poland/003/2020 | EPI_ISL_525440 |
| 2020-Jan-02 | Poland | A/chicken/Poland/004/2020 | EPI_ISL_525441 |
| 2020-Jan-06 | Poland | A/hawk/Poland/003/2020 | EPI_ISL_405813 |
| 2020-Jan-09 | Poland | A/turkey/Poland/027/2020 | EPI_ISL_525442 |
| 2020-Jan-12 | Poland | A/domestic_goose/Poland/028/2020 | EPI_ISL_525443 |
| 2020-Jan-17 | Poland | A/chicken/Poland/054/2020 | EPI_ISL_525444 |
| 2020-Jan-25 | Poland | A/turkey/Poland/079/2020 | EPI_ISL_525445 |
| 2020-Jan-27 | Poland | A/laying_hen/Poland/095/2020 | EPI_ISL_525446 |
| 2020-Jan-28 | Poland | A/turkey/Poland/096/2020 | EPI_ISL_525447 |
| 2020-Feb-20 | Poland | A/domestic_duck/Poland/219/2020 | EPI_ISL_525449 |
| 2020-Feb-20 | Poland | A/domestic_duck/Poland/221/2020 | EPI_ISL_525450 |
| 2020-Feb-21 | Poland | A/domestic_duck/Poland/222/2020 | EPI_ISL_525451 |
| 2020-Mar-01 | Poland | A/domestic_goose/Poland/274/2020 | EPI_ISL_525462 |
| 2020-Jan-16 | Germany | A/white-fronted goose/Germany-BB/AI00018/2020(H5N8) | EPI_ISL_404993 |
| 2020-Feb-06 | Germany | A/chicken/Germany-BW/AI00049/2020 | EPI_ISL_410291 |
| 2020-Mar-12 | Germany | A/chicken/Germany-SN/AI00276/2020 | EPI_ISL_415197 |
| 2020-Mar-20 | Germany | A/buzzard/Germany-SN/AI00285/2020 | EPI_ISL_417414 |
| 2020-Mar-20 | Germany | A/turkey/Germany-NI/AI00334/2020 | EPI_ISL_417415 |
| 2020-Mar-26 | Germany | A/steamer duck/Germany-SN/AI00346/2020 | EPI_ISL_419312 |
| 2020-Mar-27 | Germany | A/turkey/Germany-ST/AI00352/2020 | EPI_ISL_419314 |
| 2020-Jan-17 | Czech Republic | A/chicken/Czech Republic/1175–1/2020 | EPI_ISL_405391 |
| 2020-Feb-17 | Czech Republic | A/turkey/Czech Republic/3071/2020 | EPI_ISL_418266 |
| 2020-Jan-09 | Hungary | A/turkey/Hungary/1020_20VIR749–1/2020 | EPI_ISL_419220 |
| 2020-Mar-25 | Hungary | A/Duck/Hungary/14788/2020 | EPI_ISL_780096 |
| 2020-Mar-26 | Hungary | A/Goose/Hungary/15267/2020 | EPI_ISL_796011 |

**Appendix Table 4.** Time of the most recent common ancestor of selected node that contained each gene segment of viruses associated with H5N8 viruses isolated in Ningxia, China, October 2020*.

| | | | 95% HPD | | |
|---|---|---|---|---|---|
| Segment | Node† | tMRCA | Earliest date | Latest date | Posterior value |
| PB2 | 1 | 2020 Sep. | 2020 Aug. | 2020 Sep. | 0.9873 |
| | 2 | 2020 Jul. | 2020 May. | 2020 Sep. | 1 |
| | 3 | 2020 Aug. | 2020 Jul. | 2020 Oct. | 0.9989 |
| PB1 | 1 | 2020 Jul. | 2020 Apr. | 2020 Sep. | 1 |
| | 2 | 2020 Jul. | 2020 May. | 2020 Aug. | 0.2388 |
| PA | 1 | 2020 Aug. | 2020 Jul. | 2020 Sep. | 1 |
| | 2 | 2020 May. | 2020 Feb. | 2020 Aug. | 1 |
| | 3 | 2020 Aug. | 2020 Jul. | 2020 Sep. | 0.4015 |
| HA | 1 | 2020 Sep. | 2020 Jul. | 2020 Sep. | 1 |
| | 2 | 2020 May. | 2020 Feb. | 2020 Aug. | 1 |
| | 3 | 2020 Jun. | 2020 Apr. | 2020 Aug. | 0.4552 |
| NP | 1 | 2020 Jun. | 2020 Mar. | 2020 Aug. | 1 |
| | 2 | 2020 Aug. | 2020 Jul. | 2020 Oct. | 1 |
| NA | 1 | 2020 Aug. | 2020 Jun. | 2020 Sep. | 0.9702 |
| | 2 | 2020 Jun. | 2020 Feb. | 2020 Sep. | 1 |
| | 3 | 2020 Aug. | 2020 Jun. | 2020 Oct. | 0.5622 |
| M | 1 | 2020 Sep. | 2020 Aug. | 2020 Oct. | 0.9999 |
| | 2 | 2020 Aug. | 2020 May. | 2020 Sep. | 1 |
| | 3 | 2020 Oct. | 2020 Aug. | 2020 Oct. | 0.9767 |
| NS | 1 | 2020 Sep. | 2020 Aug. | 2020 Oct. | 1 |
| | 2 | 2020 Jul. | 2020 Apr. | 2020 Sep. | 1 |
| | 3 | 2020 Sep. | 2020 Aug. | 2020 Oct. | 0.9613 |

*HPD, highest posterior density; PB2, polymerase basic 2; PB1, polymerase basic 1; PA, polymerase acidic; HA, hemagglutinin; NP, nucleoprotein; M, matrix; NS, nonstructural; tMRCA, time of the most recent common ancestor.

†Nodes marked in the maximum clade credibility phylogenetic trees (Appendix Figure 2).

**Appendix Table 5.** Molecular characterization of H5N8 viruses isolated in Ningxia, October 2020, and the H5N8 virus that infected humans in Russia, December 2020*

| Protein | Position | Ningxia b1 isolates | Ningxia b2 isolate | A/Astrakhan/3212/2020(H5N8) |
|---|---|---|---|---|
| HA | S133A | + | + | + |
| (H5 numbering)† | T156A | + | + | + |
| | T188I | - | + | - |
| | V210I | - | + | - |
| | Q222L | - | - | - |
| | G224S | - | - | - |
| PB2‡ | Q591K | - | - | - |
| | E627K | - | - | - |
| | D701N | - | - | - |
| M1§ | N30D | + | + | + |
| | I43M | + | + | + |
| | T215A | + | + | + |
| NS§ | P42S | + | + | + |
| | 80–84 deletion | NO | NO | NO |
| | L98F | + | + | + |
| | I101M | + | + | + |

*HA, hemagglutinin; PB2, polymerase basic 2; M, matrix; NS, nonstructural; Ningxia b1 isolates, the twelve clade 2.3.4.4b1 H5N8 viruses isolated in Ningxia, October 2020; Ningxia b2 isolate, A/common teal/Ningxia/105/2020(H5N8), the clade 2.3.4.4b2 H5N8 virus isolated in Ningxia, October 2020; +, mutation; –, no mutation.

†S133A, T156A, T188I, V210I, Q222L, and G224S mutations in HA have been associated with increased binding to human-like receptor (α-2–6 sialic acid).

‡Q591K, E627K, and D701N mutations have been associated with increased adaptation of avian influenza virus in mammalian host.

§N30D, I43M, T215A, P42S, 80–84 deletion, L98F, and I101M mutations have been associated with increased virulence in mice.

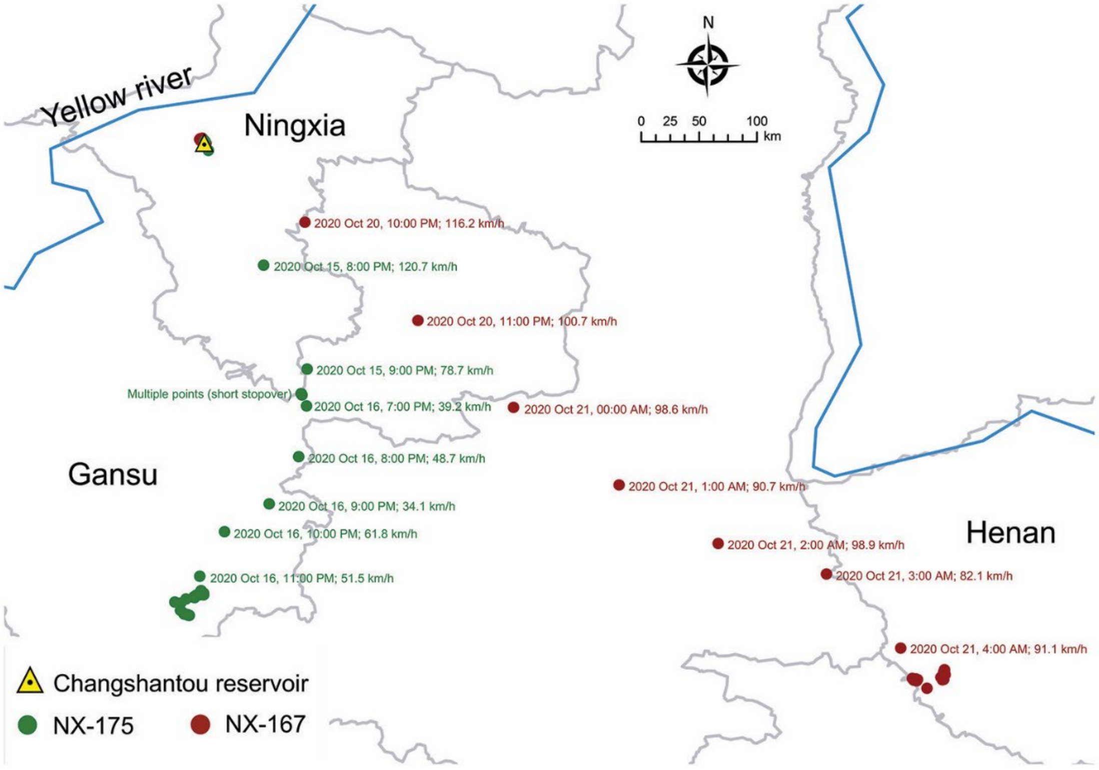


**Appendix Figure 1.** During October 15–30, 2020, movements of 2 successfully satellite-tracked mallards (*Anas platyrhynchos*). Time and flight speed of every moving point during the migration of the 2 mallards is displayed. The sampling site (Changshantou Reservoir) is indicated.

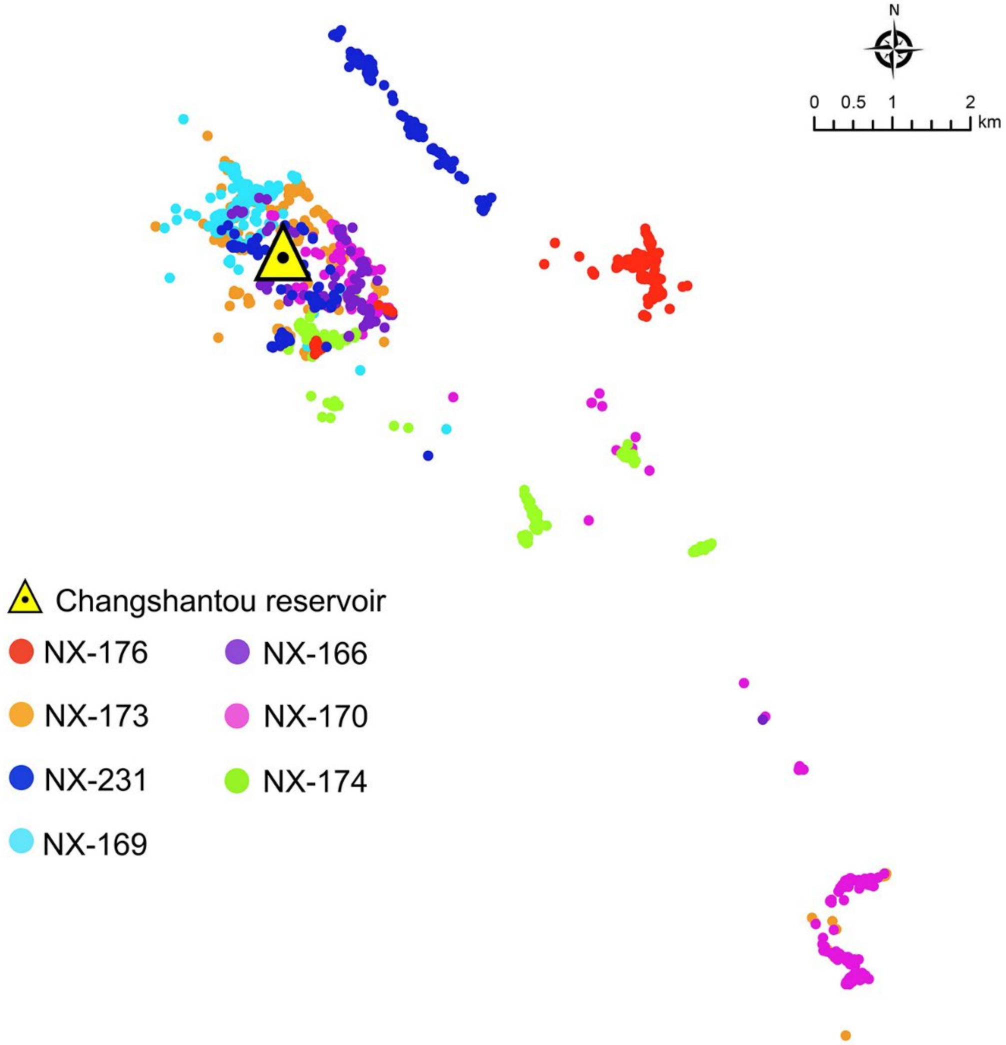


**Appendix Figure 2.** During October 15–30, 2020, movements of 7 successfully satellite-tracked mallards. Movements are represented by different colors. The sampling site at Changshantou Reservoir is indicated.

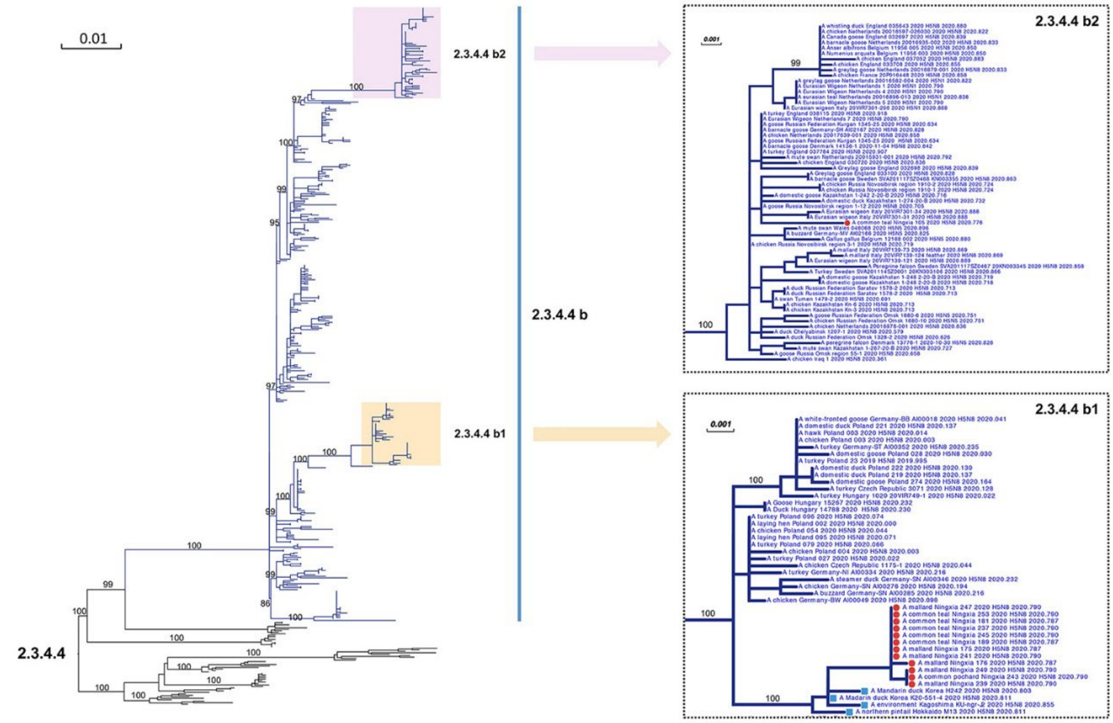


**Appendix Figure 3**. Maximum-likelihood phylogenetic trees for hemagglutinin genes of avian influenza A(H5N8) viruses isolated in Ningxia, China, 2020, and comparison with clade 2.3.4.4 reference isolates. Dark blue lines indicate viruses that belonged to clade 2.3.4.4b, and black lines indicate viruses that belonged to clade 2.3.4.4a, c–h. Subtrees marked with orange and purple backgrounds indicate subclade 2.3.4.4b1 and subclade 2.3.4.4b2, respectively. The 2 subtrees indicated in the 2 boxes on the right show our Ningxia H5N8 isolates (red circles) and H5N8 viruses isolated from South Korea and Japan during October and November 2020 (blue squares). UFBoot support values of the major branch are indicated. Numbers along branches are bootstrap values. Scale bars indicate nucleotide substitutions per site.

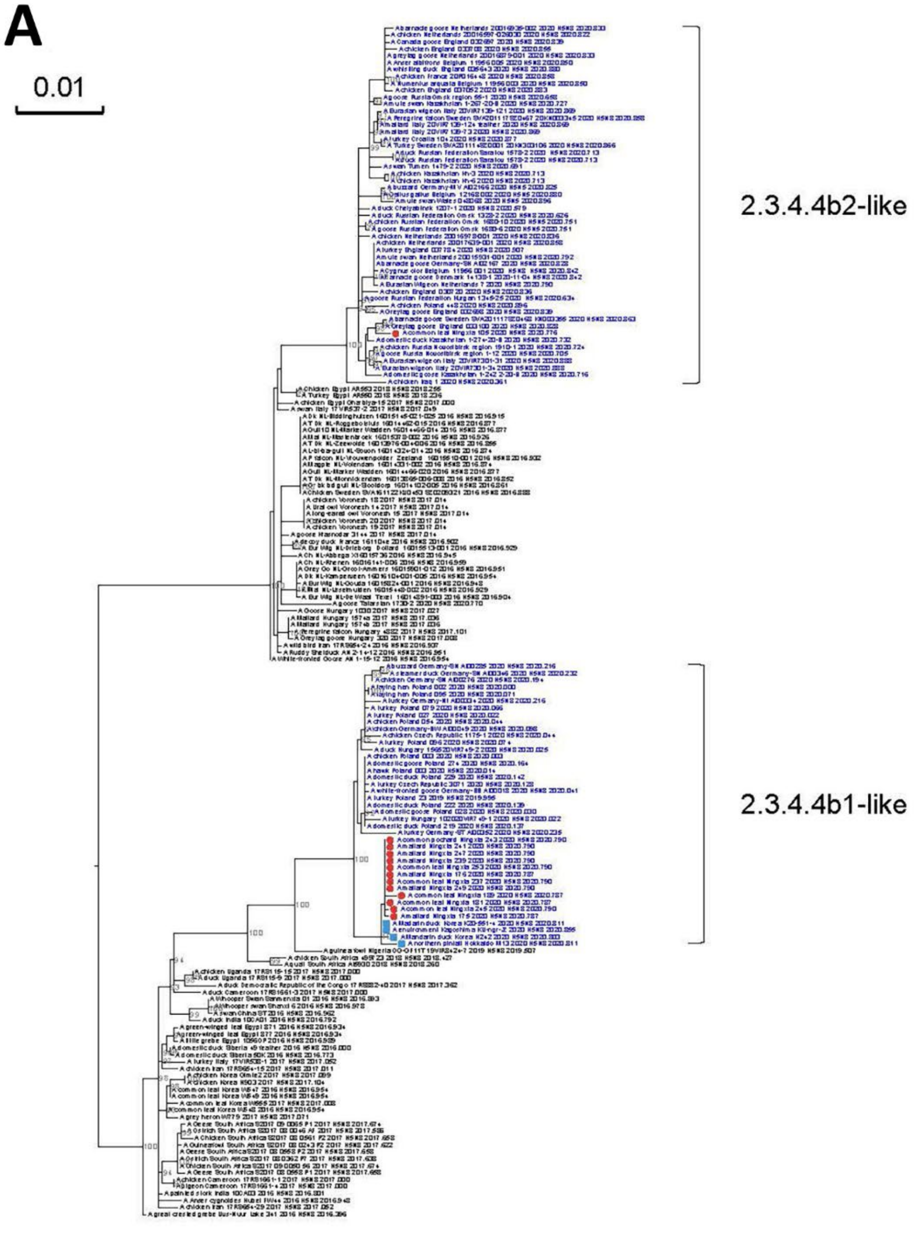
A
0.01
2.3.4.4b2-like
2.3.4.4b1-like

**B**

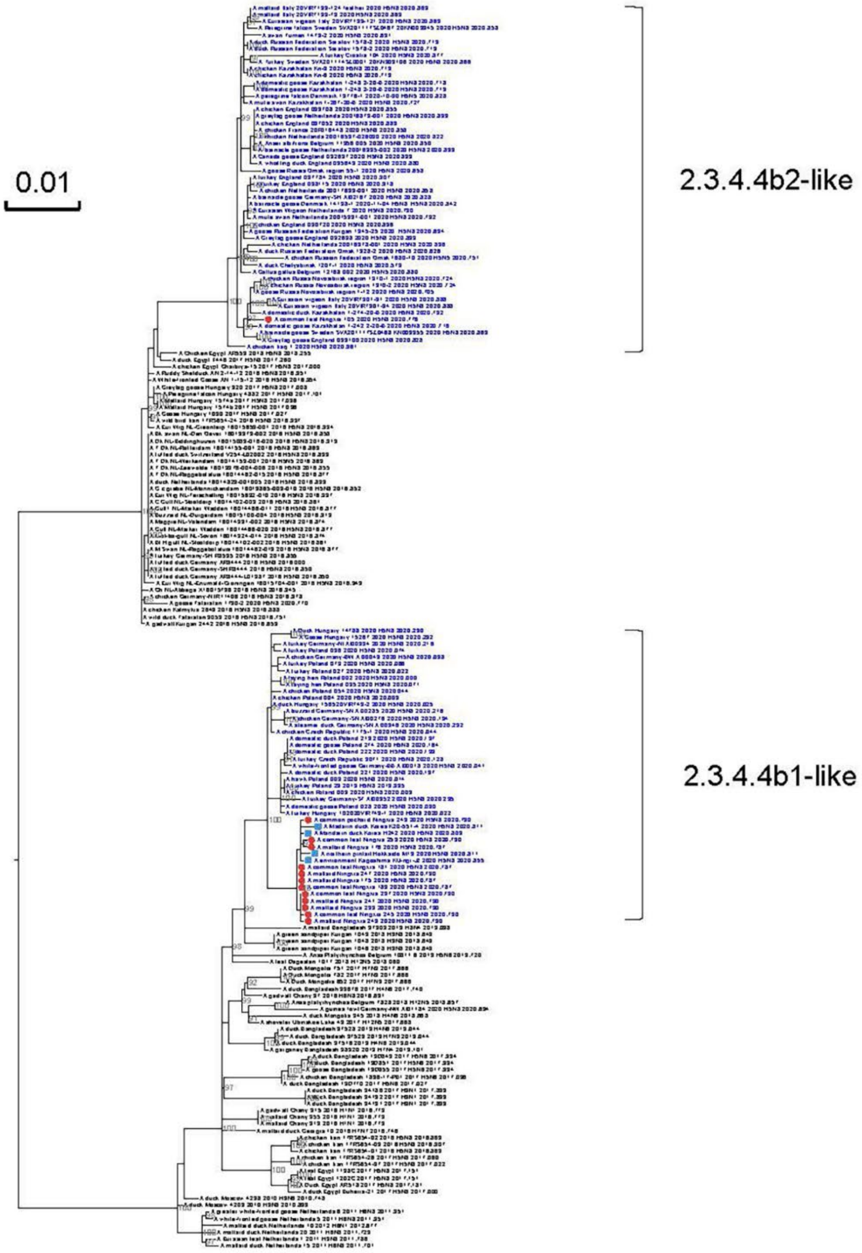

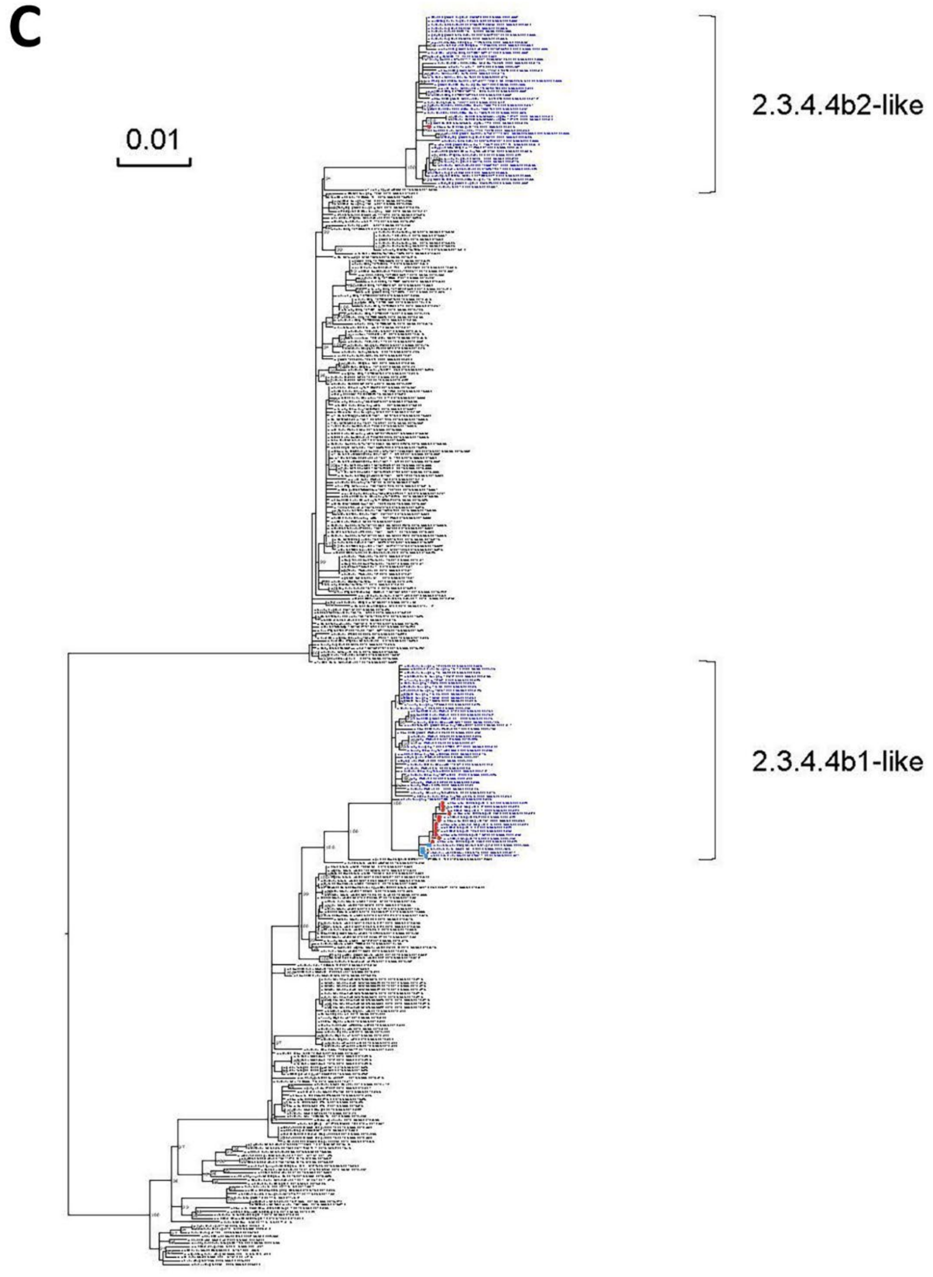
C
0.01
2.3.4.4b2-like
2.3.4.4b1-like

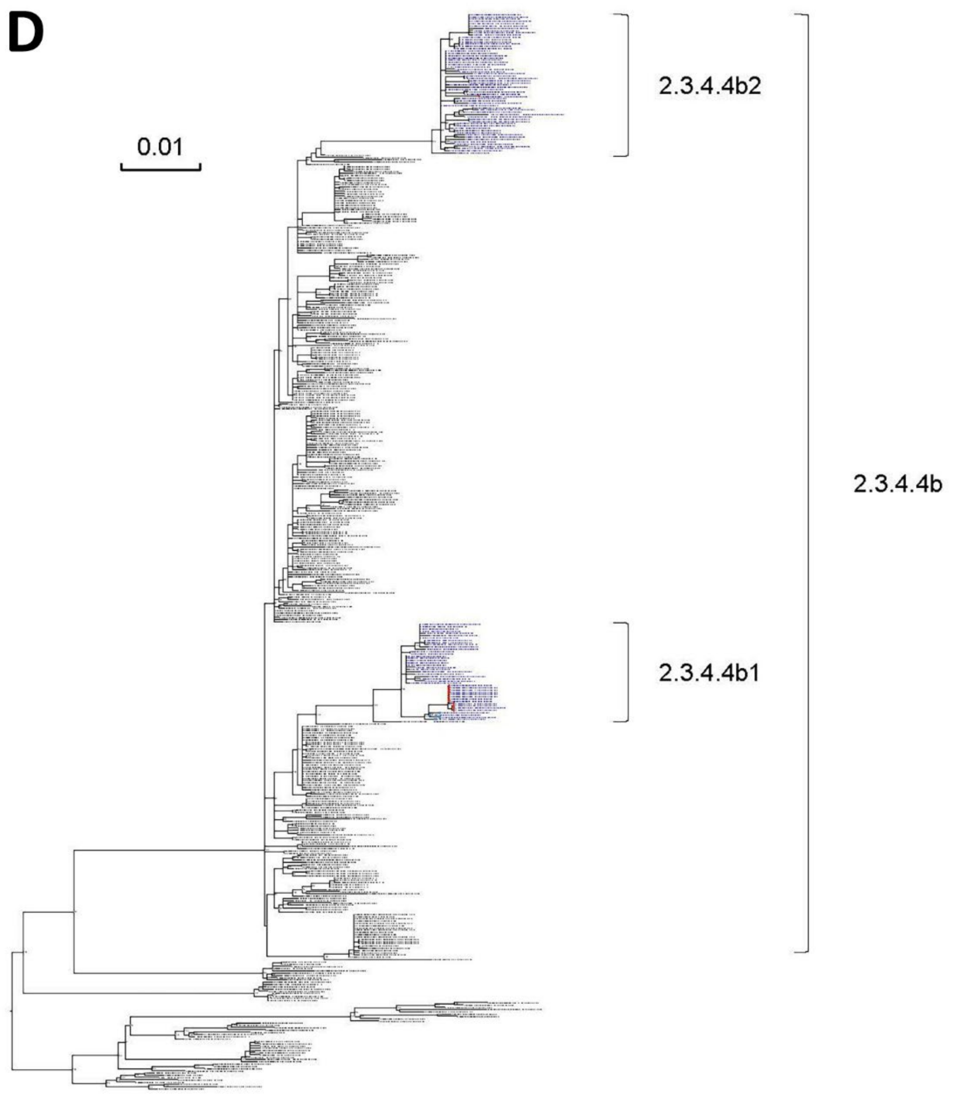
D
0.01
2.3.4.4b2
2.3.4.4b
2.3.4.4b1

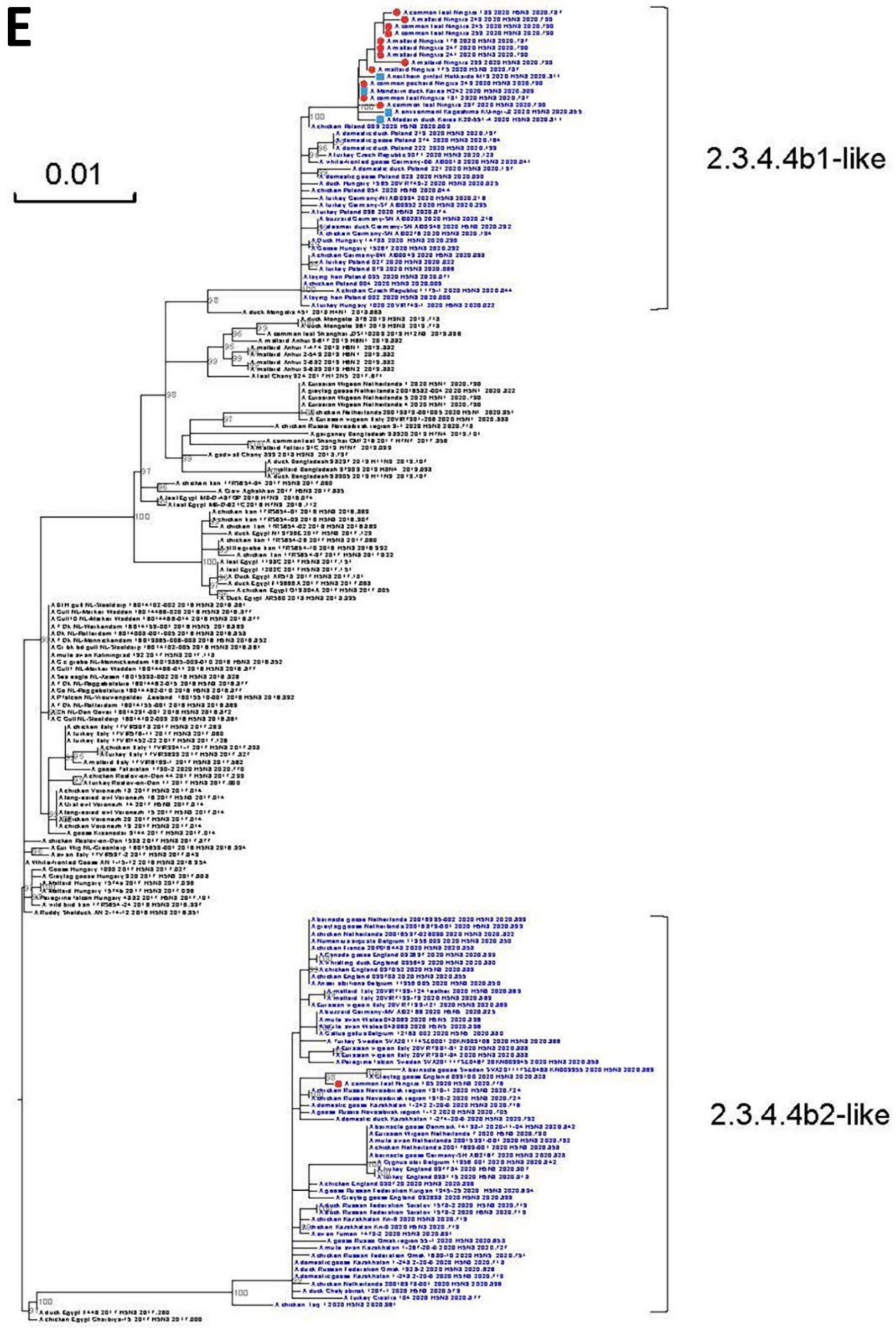
E
0.01
2.3.4.4b1-like
2.3.4.4b2-like

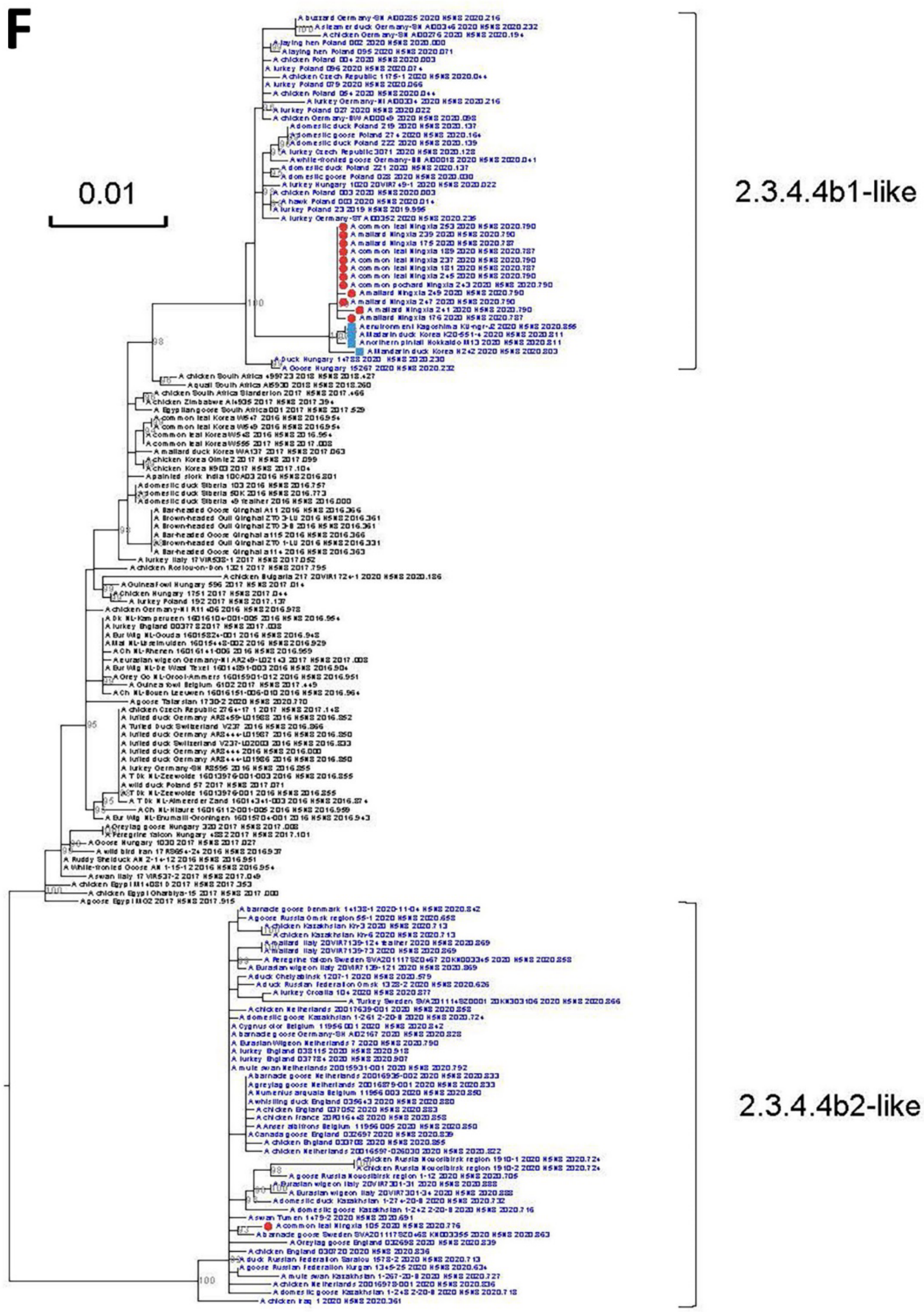
F
0.01
2.3.4.4b1-like
2.3.4.4b2-like

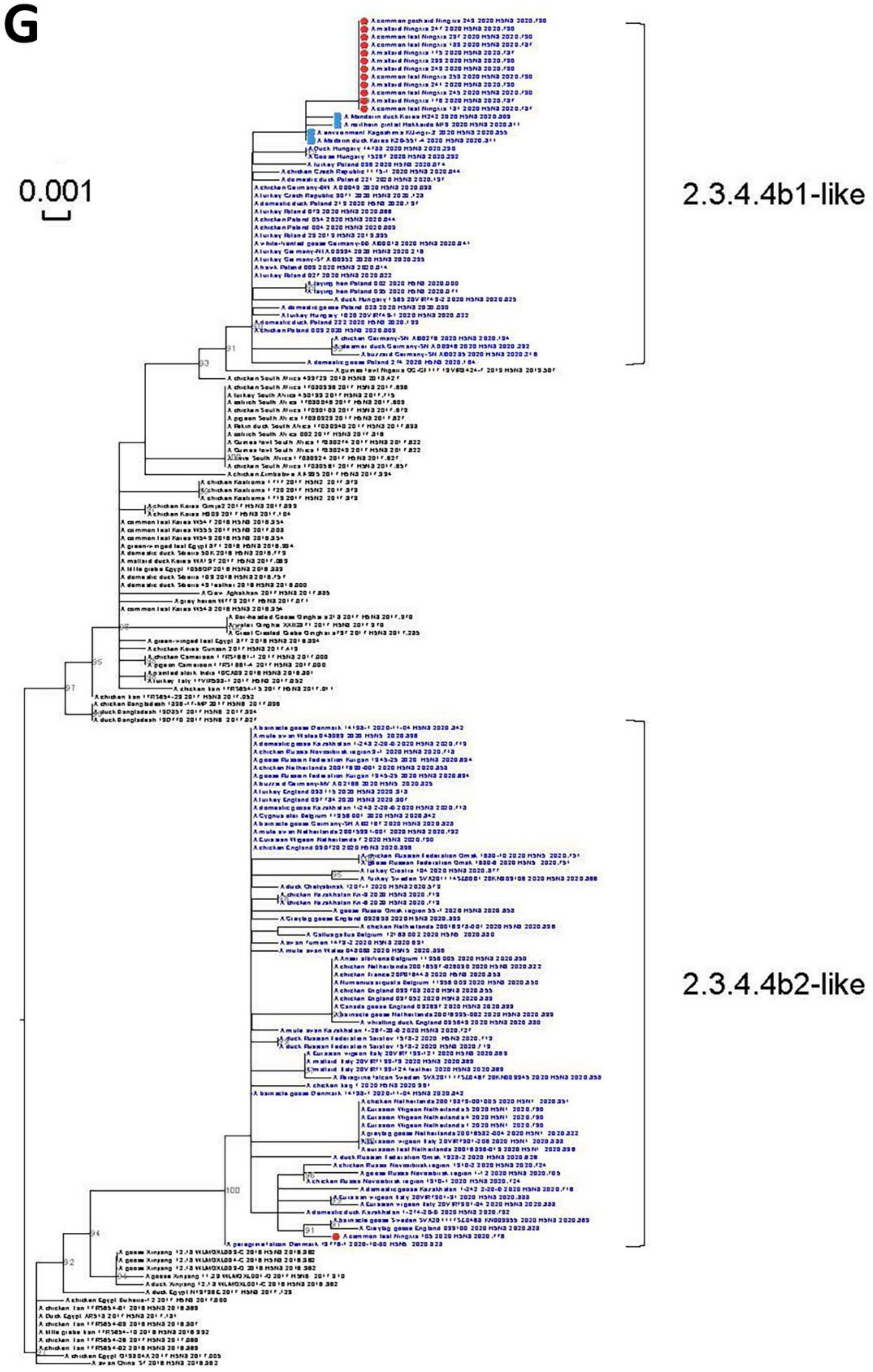
G
0.001
2.3.4.4b1-like
2.3.4.4b2-like

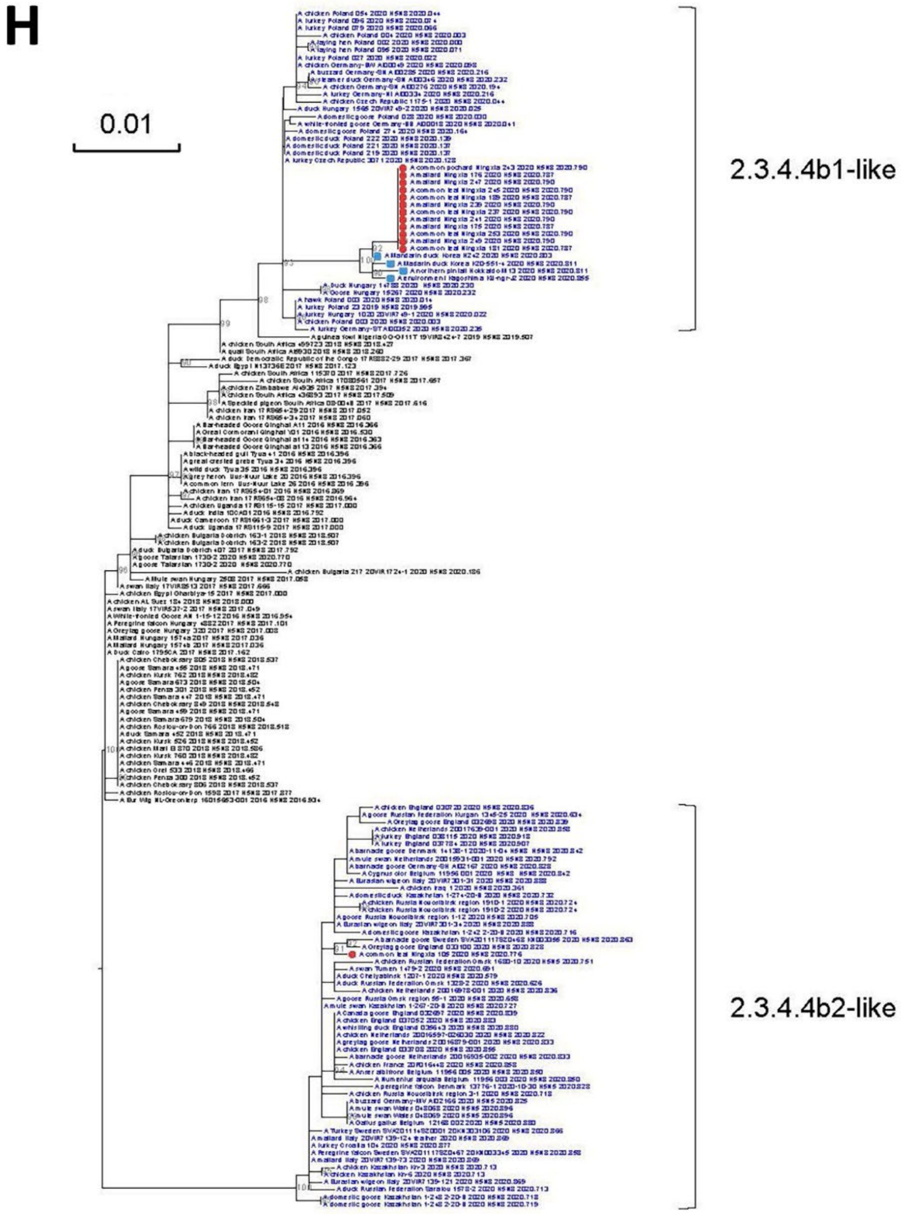


**Appendix Figure 4.** Maximum-likelihood phylogenetic trees. Our Ningxia H5N8 isolates are marked with red circles, the H5N8 viruses from South Korea and Japan isolated in October and November 2020 are marked with blue squares, and the clade 2.3.4.4b viruses isolated in 2020 are shown in blue. UFBoot support values >90 are shown. A) polymerase basic 2; B) polymerase basic 1; C) polymerase acidic; D) hemagglutinin; E) nucleoprotein; F) neuraminidase; G) matrix; H) nonstructural. Values along branches are bootstrap values. Scale bars indicate nucleotide substitutions per site.

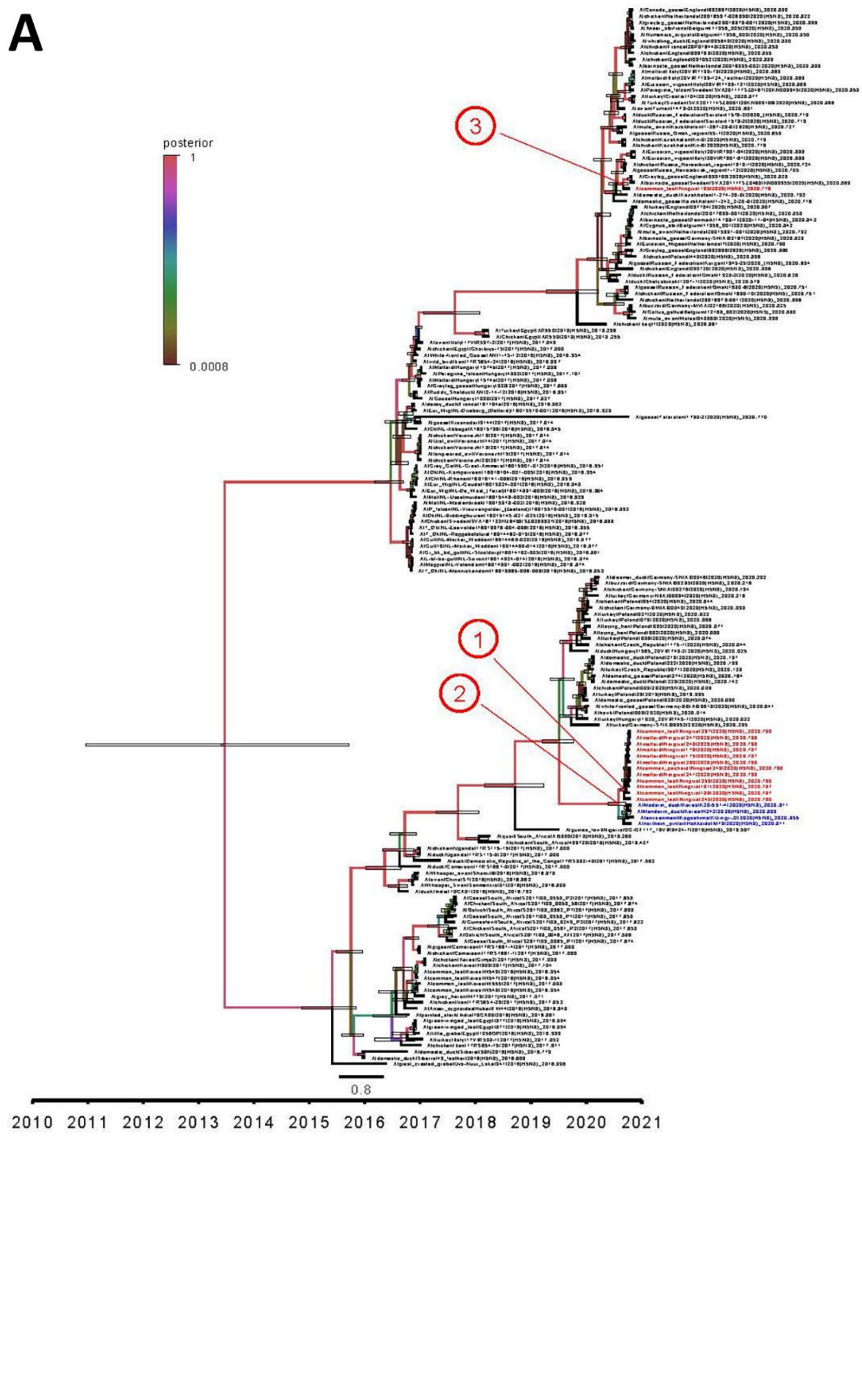
A
posterior
1
0.0008
3
1
2
0.8
2010 2011 2012 2013 2014 2015 2016 2017 2018 2019 2020 2021

**B**

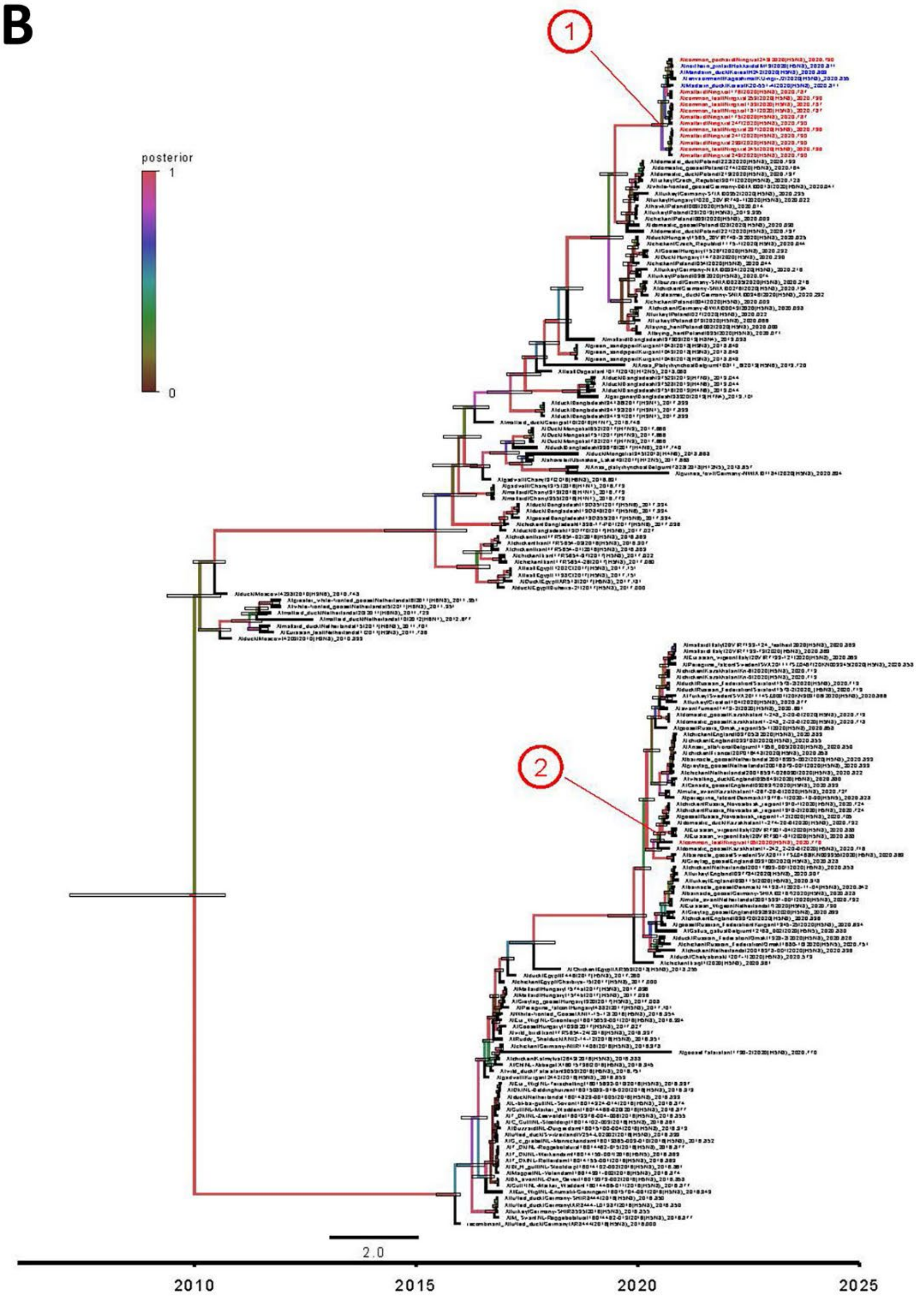

C

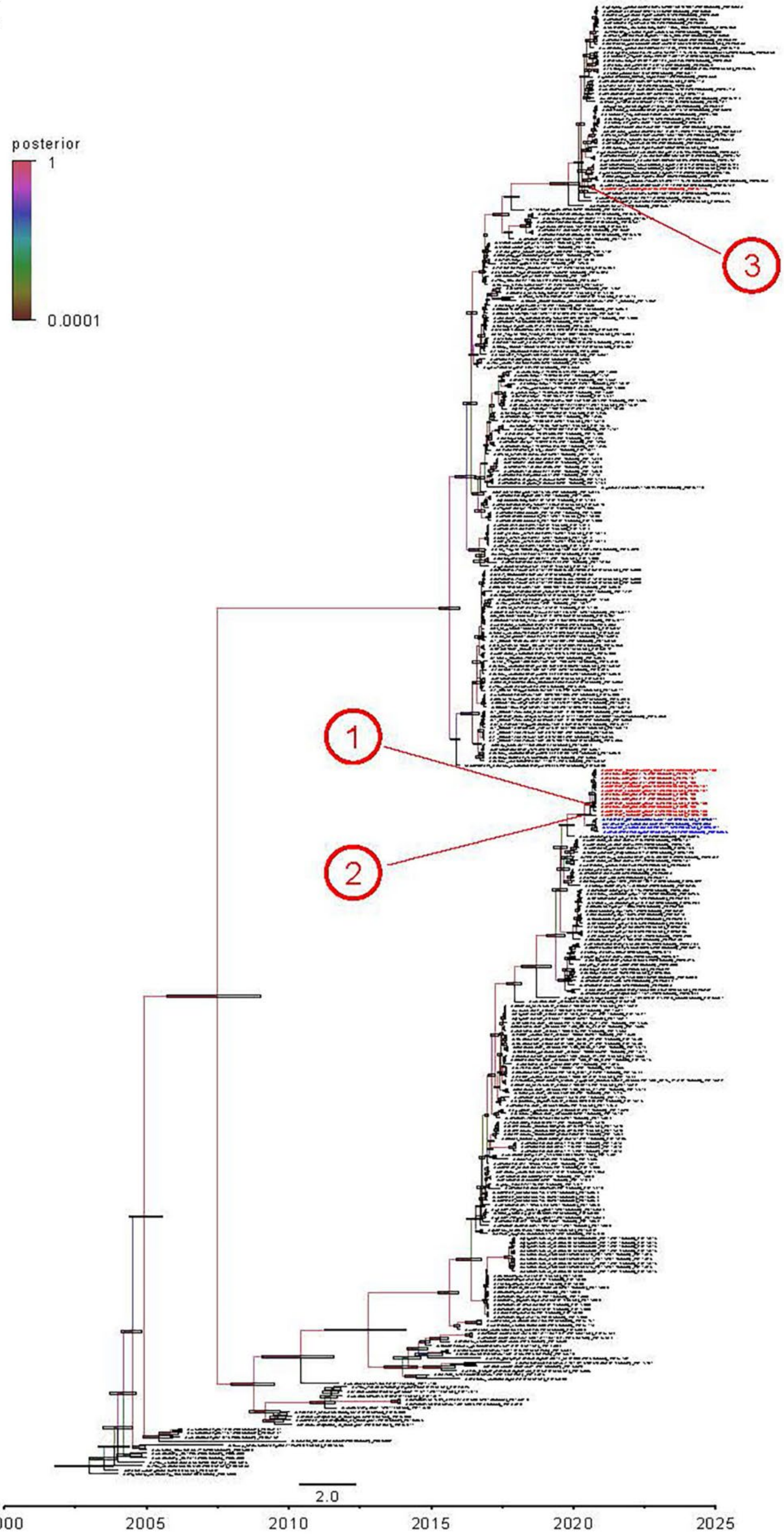

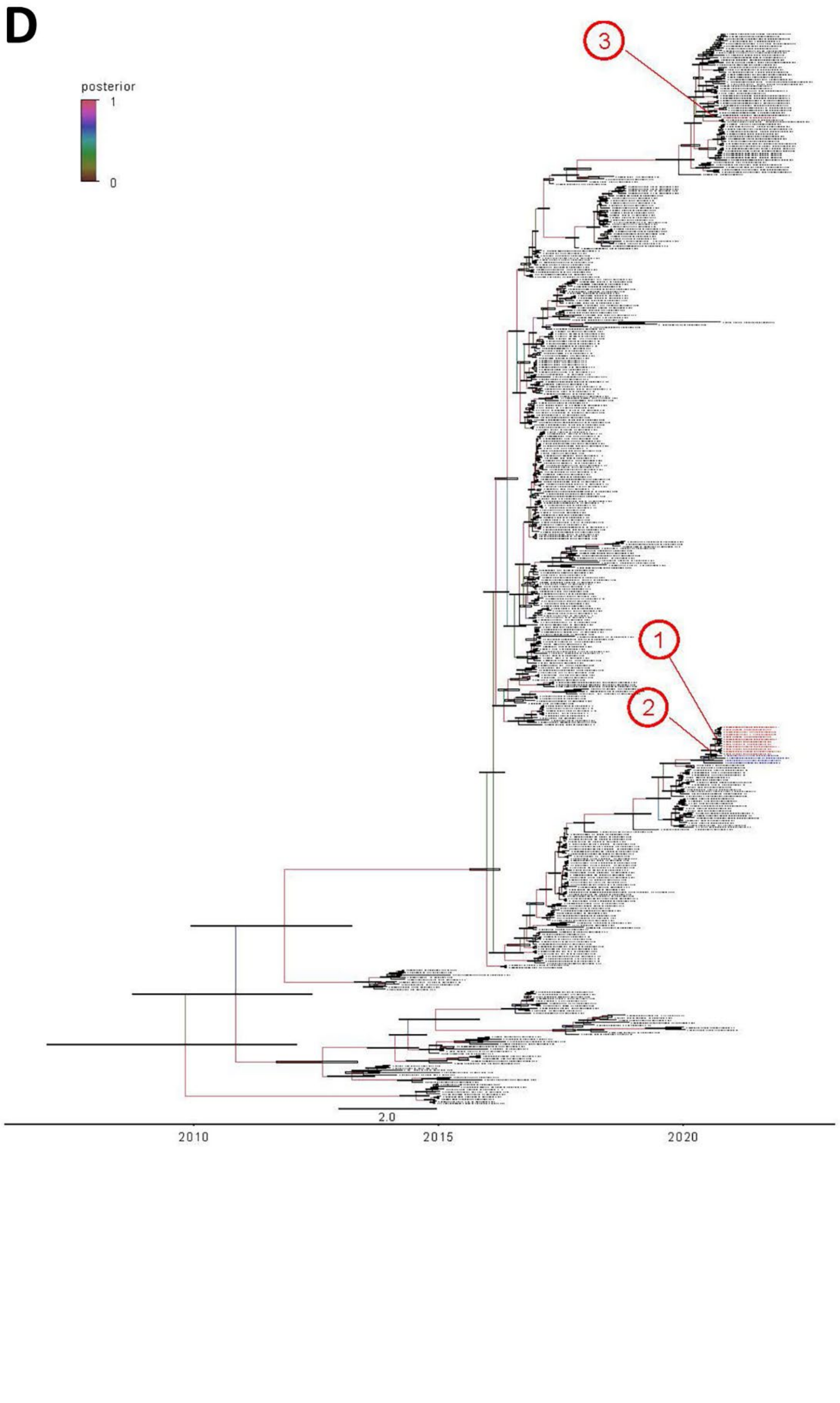
D
posterior
1
0
3
1
2
2.0
2010
2015
2020

**E**

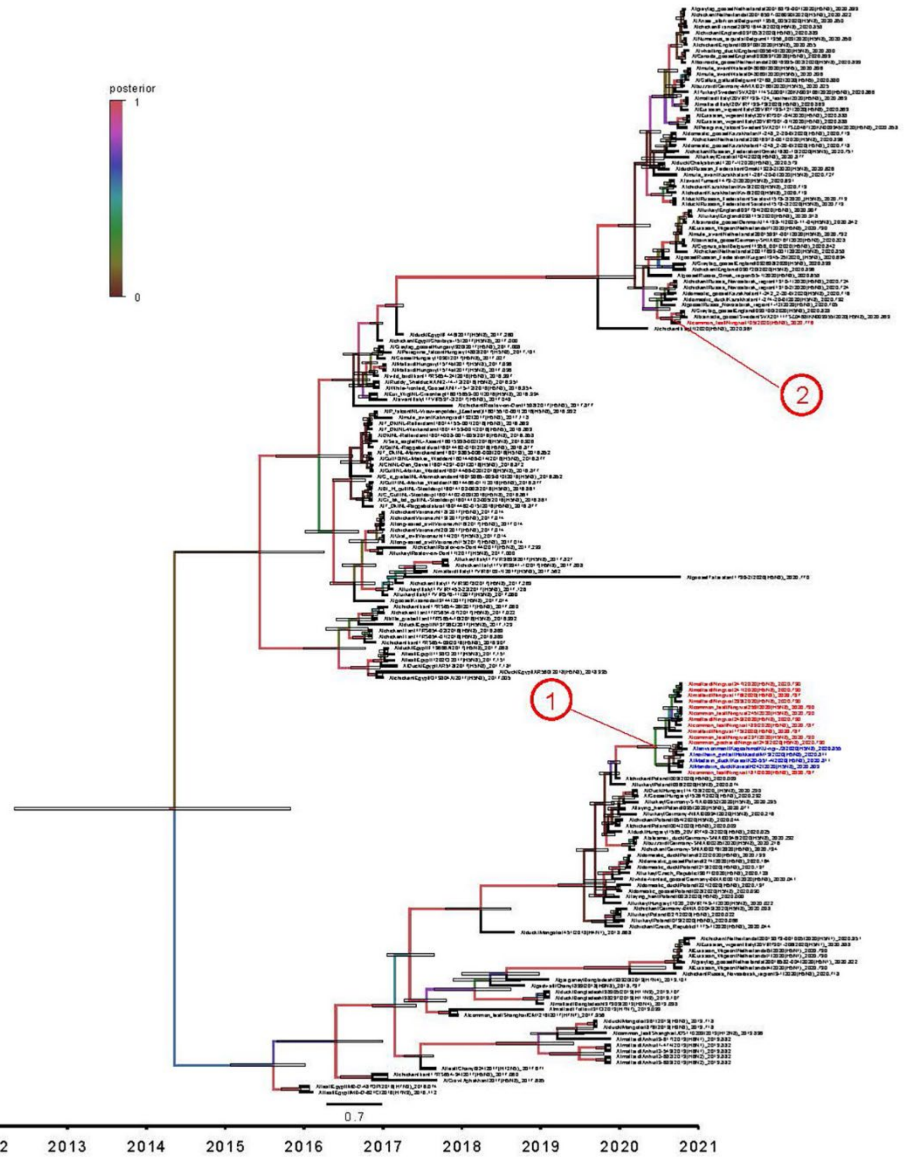
posterior
1
0
1
2
0.7
2012
2013
2014
2015
2016
2017
2018
2019
2020
2021

F

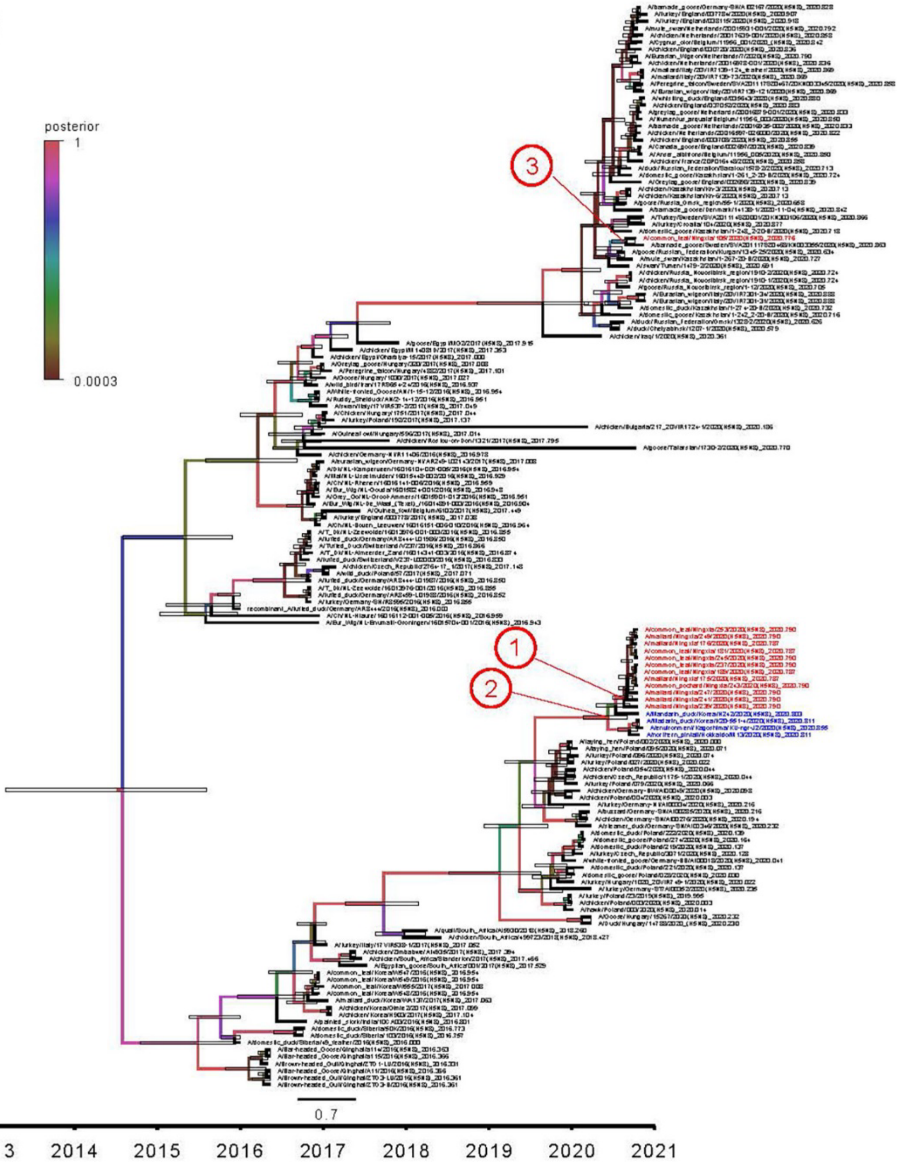
posterior
1
0.0003
3
1
2
0.7
2013
2014
2015
2016
2017
2018
2019
2020
2021

G

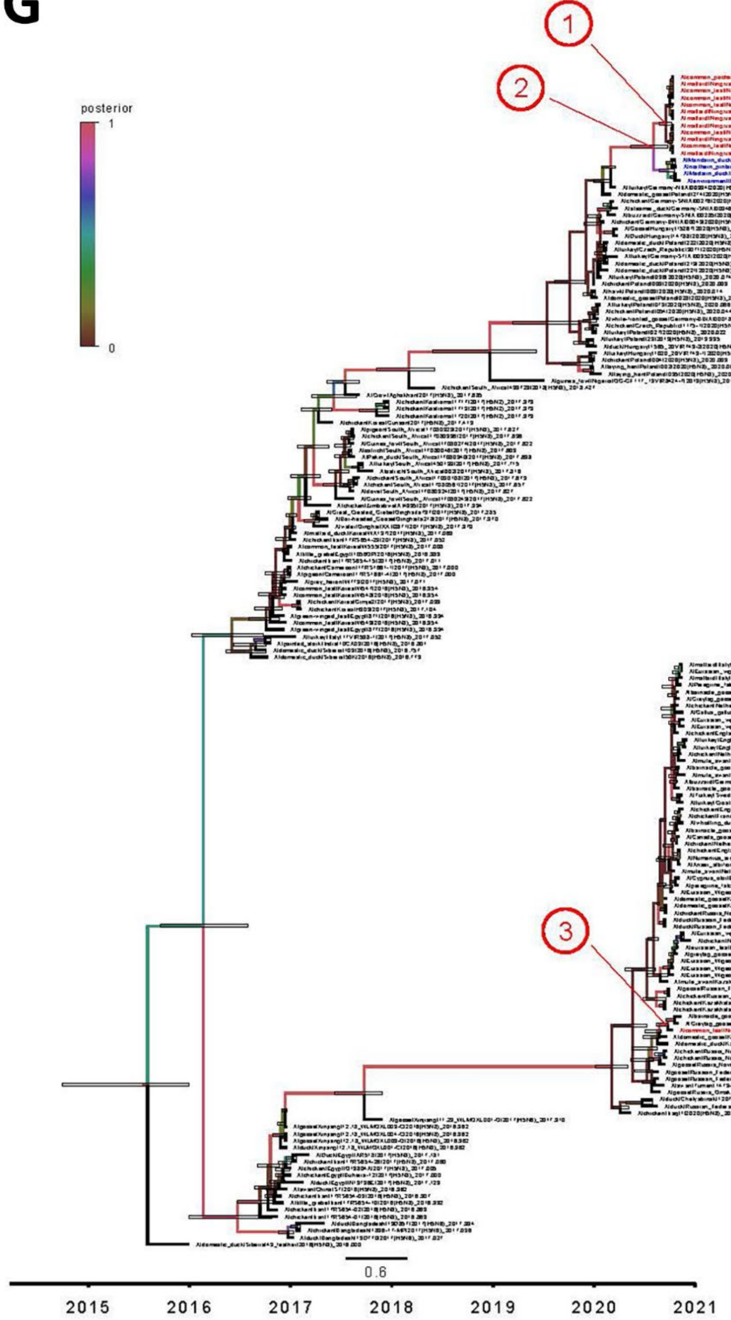

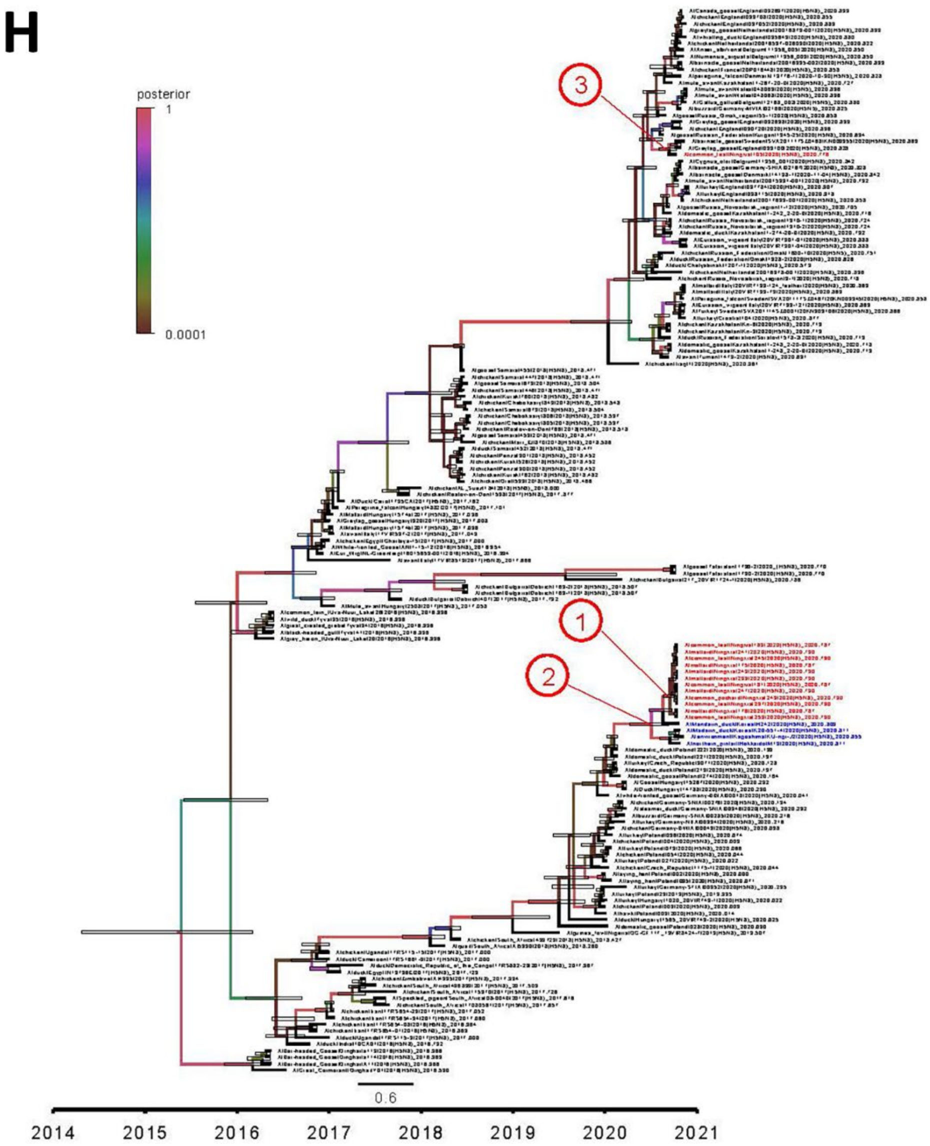


**Appendix Figure 5.** Maximum clade credibility phylogenetic trees (years on the horizontal axis) of each gene segment of the viruses associated with the H5N8 viruses isolated in Ningxia, October 2020. The Ningxia H5N8 isolates are shown in red, and the H5N8 viruses isolated in South Korea and Japan during October and November 2020 are shown in blue. The horizontal bars indicate the 95% highest posterior density (HPD) intervals of the most recent common ancestor. A) polymerase basic 2; B) polymerase basic 1; C) polymerase acidic; D) hemagglutinin; E) nucleoprotein; F) neuraminidase; G) matrix; H) nonstructural. Values along branches are bootstrap values. Scale bars indicate nucleotide substitutions per site.
